# Investigating the continuous synthesis of a nicotinonitrile precursor to nevirapine

**DOI:** 10.3762/bjoc.9.292

**Published:** 2013-11-20

**Authors:** Ashley R Longstreet, Suzanne M Opalka, Brian S Campbell, B Frank Gupton, D Tyler McQuade

**Affiliations:** 1 Department of Chemistry and Biochemistry, Florida State University, 95 Chieftan Way, Tallahassee, FL 32306, United States; 2 Department of Chemistry, Virginia Commonwealth University, 1001 West Main Street, P.O. Box 842006 Richmond, Virginia 23284, United States

**Keywords:** continuous, flow chemistry, HIV, Knoevenagel, nevirapine, nicotinonitriles

## Abstract

2-Chloro-3-amino-4-picoline (CAPIC) is a strategic building block for the preparation of nevirapine, a widely-prescribed non-nucleosidic reverse transcriptase inhibitor for the treatment of HIV-infected patients. A continuous synthesis to the bromo derivative of a CAPIC intermediate, 2-bromo-4-methylnicotinonitrile, that terminates in a dead-end crystallization is described. The route uses inexpensive, acyclic commodity-based raw materials and has the potential to enable lower cost production of nevirapine as well as other value added structures that contain complex pyridines. The route terminates in a batch crystallization yielding high purity CAPIC. This outcome is expected to facilitate regulatory implementation of the overall process.

## Introduction

**Figure 1:**
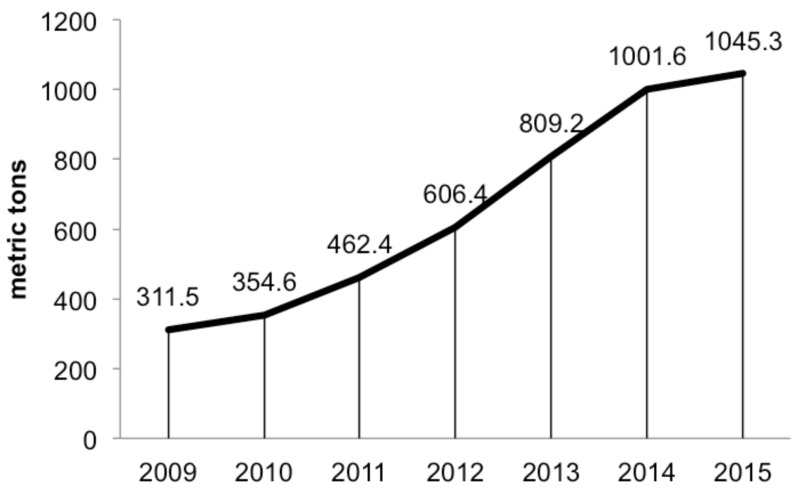
The estimated demand of for nevirapine until 2015 [6].

Nevirapine (**3**) was the first commercially available non-nucleoside reverse transcriptase inhibitor (NNRTI), and has remained an important medicine in the management of human immunodeficiency virus (HIV) [1–2]. Nevirapine combined with lamivudine (3TC) and azidothymidine (AZT) or tenofovir (TDF) is one of the preferred first-line combination drug therapies recommended by the World Health Organization (WHO) [3–5]. WHO initiatives are expected to increase the demand for nevirapine over the next 10 years (Figure 1) [6]. Although several viable NNRT substitutes for nevirapine are available, nevirapine manufacturing requirements will remain high because clinicians are reluctant to change treatment once a successful combination therapy is identified and many remain healthy with the nevirapine based combinations. Furthermore, the recent development of an extended release dosage form of nevirapine that enables once a day administration is expected to further increase market demand [6–8]. The high demand coupled with the financial burden associated with long-term HIV treatments has resulted in shortages and patients opting to reduce dosing which increases development of resistant strains [9–12]. This confluence of increased demand and cost provides an opportunity to reevaluate both the chemistry as well as the manufacturing platforms by which this drug can be produced.

**Scheme 1:**
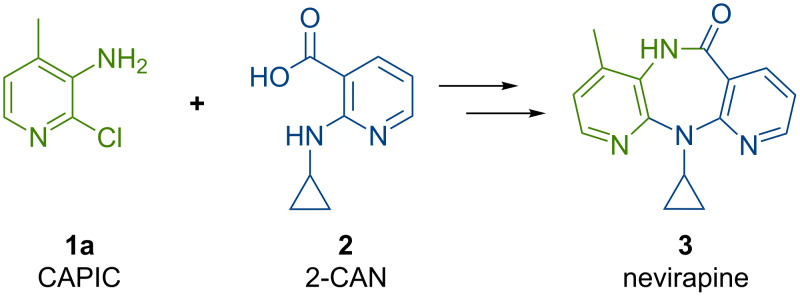
Commercial building blocks to nevirapine.

The two key Food and Drug Administration (FDA) registered starting materials in the commercial nevirapine process are 2-chloro-3-amino-4-picoline (CAPIC) (**1a**) and 2-cyclopropylaminonicotinic acid (2-CAN) (**2**) (Scheme 1) [13]. The CAPIC process comprises approximately 64% of the total production cost. Based on our previous experience with the development of the current commercial batch processes for nevirapine [13] and its pyridine precursors [14], we have started a program to define lower costs nevirapine processes. After a cost of goods analysis, we have come to the conclusion that the most promising cost saving path forward is through the use of acyclic, commodity-based starting materials in the assembly of the active pharmaceutical ingredient (API) (analysis will be included on future publications). We hypothesized that by both reducing the cost of goods via chemistry changes and reducing the unit operations the most significant cost reduction could be achieved. Herein, we demonstrate a proof of concept flow synthesis of the key intermediate used to produce the bromo derivative of the CAPIC precursor, 2-bromo-4-methylnicotinonitrile (**6b**). The synthesis telescopes three steps using substantially less expensive starting materials.

Flow or continuous chemistry is alternative to batch chemistry where reactions are performed by passing reagents through devices containing small-dimensional channels as opposed to using batch reactors [15–17]. Flow reactors are particularly advantageous in multistep syntheses where telescoping steps avoids isolation of dangerous and/or unstable intermediates and reduces solvent usage and waste production incurred through intermediate purifications [18–27]. The large surface to volume ratios found in the small channels allow for more efficient mixing and heat transfer often resulting in shorter contact times [28–29]. Consequently, flow chemistry allows chemists to expand their window of process operability by working at elevated temperatures and pressures to increase reaction rates and decrease catalyst loadings [30–33]. Unlike scaling-up batch reactions, which requires additional optimization, scaling-up flow processes only requires implementing multiple reactors to work in parallel [29].

**Scheme 2:**
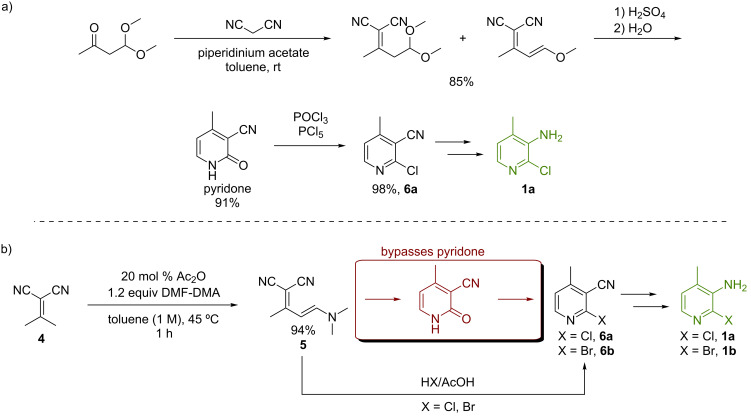
a) Current commercial process to CAPIC and b) newly developed batch synthesis to CAPIC and its bromo derivative.

We have recently developed a method to synthesize polysubstituted 2-halonicotinonitriles in high yields via enamine intermediates **5** by reacting alkylidene malononitriles in the presence of acetic anhydride with *N*,*N*-dimethylformamide dimethyl acetal (DMF-DMA) [34]. Previous attempts to synthesize the nicotinonitriles via enamines resulted in poor yields due to dimerization of the starting alkylidene malononitrile [35–37]. A high yield enamine approach allows us to begin the synthesis from the commodity chemicals (acetone and malononitrile) and bypass the pyridone intermediate used in the original CAPIC synthesis (Scheme 2a) by effecting the ring closure under Pinner reaction conditions (Scheme 2b) [14].

**Scheme 3:**
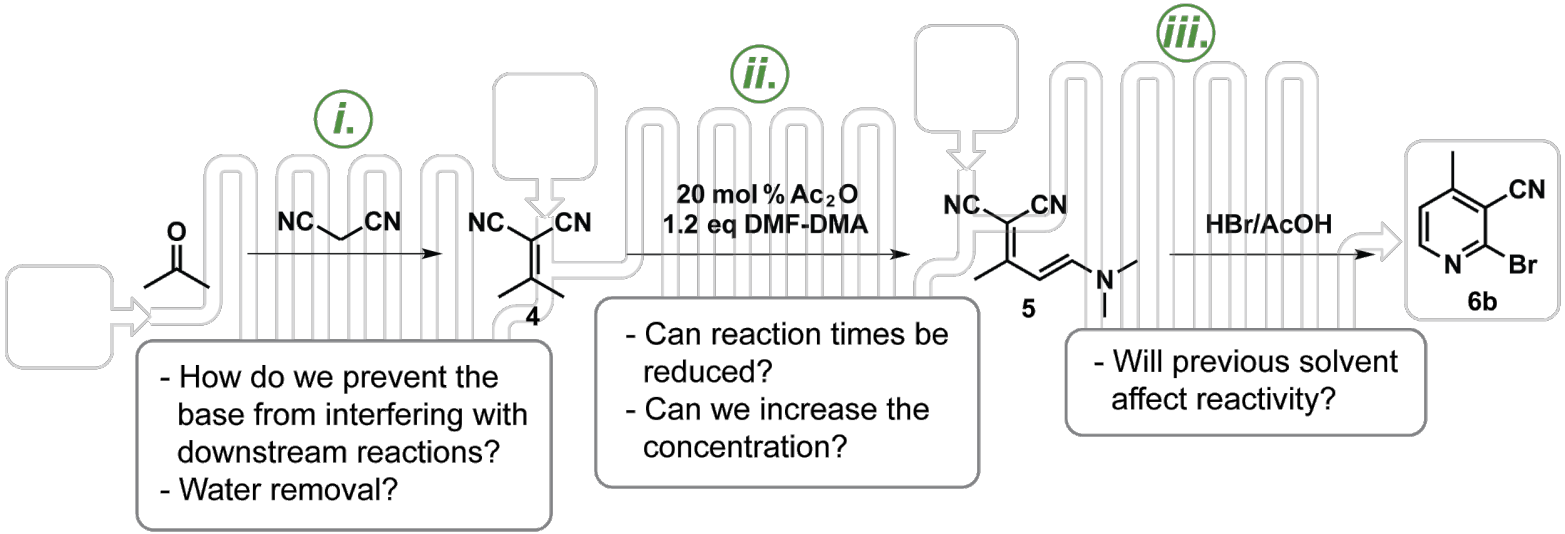
Proposed synthesis to 2-bromo-4-methylnicotinonitrile using a continuous approach with the considerations for each reaction. i. Knoevenagel condensation to produce the isopropylidenemalononitrile (**4**). ii. Reaction of the isopropylidenemalononitrile with DMF-DMA to produce an enamine (**5**). iii. Dead end cyclization to the desired 2-bromo-4-methylnicotinonitrile (**6b**) using HBr.

We set out to investigate the possibility of performing a continuous synthesis of 2-bromo-4-methylnicotinonitrile starting from acetone and malononitrile (Scheme 3) using the Vapourtec R series reactor system [38]. The batch synthesis commences with a Knoevenagel reaction condensing malononitrile and acetone catalyzed by aluminum oxide producing isopropylidenemalononitrile (**4**) [39–40]. The penultimate enamine **5** results by treating **4** with DMF-DMA in the presence of acetic anhydride, and ultimately 2-bromo-4-methylnicotinonitrile (**6b**) is produced after **5** is treated with HBr in acetic acid. Transferring the batch synthesis into a semi-continuous process requires one to consider solvent exchanges and byproducts that might complicate downstream operations.

We immediately recognized the water formed in the Knoevenagel condensation would quench the DMF-DMA in the second step. In addition, our batch Knoevenagel condensation was base catalyzed and we discovered that base increased dimer byproducts in the enamine step. Therefore, we chose to employ a solid basic reagent that would simultaneously catalyze the Knoevenagel reaction and confine the reagent to the first step, as well as a solid desiccant to remove the water. Previously we have used solid catalysts and/or solid reagents in a number of continuous processes [41–44]. Another challenge was the need to increase the rate of enamine **5** formation. Under some conditions, the enamine step required up to 24 hours [34]. Factoring these and other considerations, we designed the process shown in Scheme 3 with a summary of considerations for each step.

## Results and Discussion

**Figure 2:**
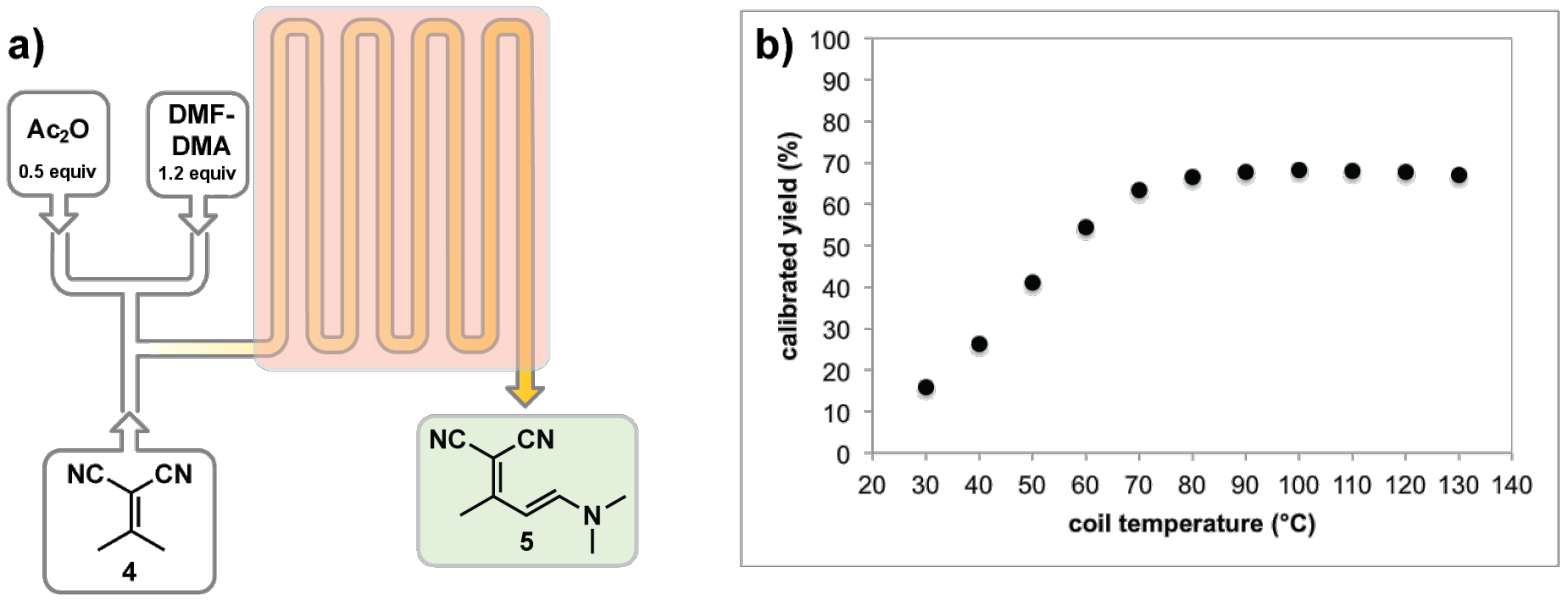
a) Flow scheme to produce the enamine intermediate **5** from isopropylidenemalononitrile (**4**) (see Supporting Information File 1 for details). b) Coil temperature effect on yield at a 0.10 M reaction concentration with a 2 min residence time.

We began our investigation by optimizing the enamine formation (**5**, Figure 2) because we predicted that success with this central step would help define the flanking reactions. Initial batch studies revealed that the reaction occurred rapidly (1 h) in toluene (1.0 M) heated to 45 °C to produce **4** in 94% yield [34], but the product precipitated and these conditions were rejected to avoid reactor clogging. Based on literature precedent and our own screening, we discovered that DCM solubilized **4**, **5**, and **6b** (Scheme 3). In batch, use of DCM would require non-traditional glassware because temperatures exceeding the standard boiling point at atmospheric pressure were required to avoid unwanted dimer formation unless low reaction concentrations (0.10 M) were used. A flow reactor is ideal for performing reactions well outside of normal operating conditions and we pushed forward seeking high temperature conditions using DCM [45].

When screening conditions, we initially investigated the effect temperature had on the reaction at a 0.10 M concentration (Figure 2b). Placing backpressure regulators after the heated coil allowed the temperature of the reactor coil to be raised far above the boiling point of DCM. As shown in Figure 2b, increasing the temperature to 80 °C provided 67% yield with a 2 min residence time. We then examined reaction concentrations to reduce the volume of DCM (Table 1). We were not only able to increase the concentration to 1.0 M by heating to 95 °C, but were also able to increase yields to >93% with a 2 min residence time. In comparison, our batch method with similar concentration conditions in toluene was complete in 1 h with 94% yield [34]. These flow conditions were high yielding in one thirtieth of the reaction time. This example underscores the benefit of operating outside of normal process windows [45]. Attempts to increase the reaction concentration beyond 1.0 M led to reactor clogging due to the limited of solubility of **5**.

**Table 1:** Concentration screen for enamine formation.

Entry	Reaction concentration (M)	Residence time (min)	Coil temperature (°C)	Yield (%)^a^

1	0.10	2	100	68
2	0.20	2	95	93
3	0.40	2	95	96
4	0.60	2	95	98
5	0.80	2	95	97
6	0.98	2	95	97

^a^Determined by GC analysis using mesitylene as an internal standard. See Figure 2a for flow scheme.

**Scheme 4:**
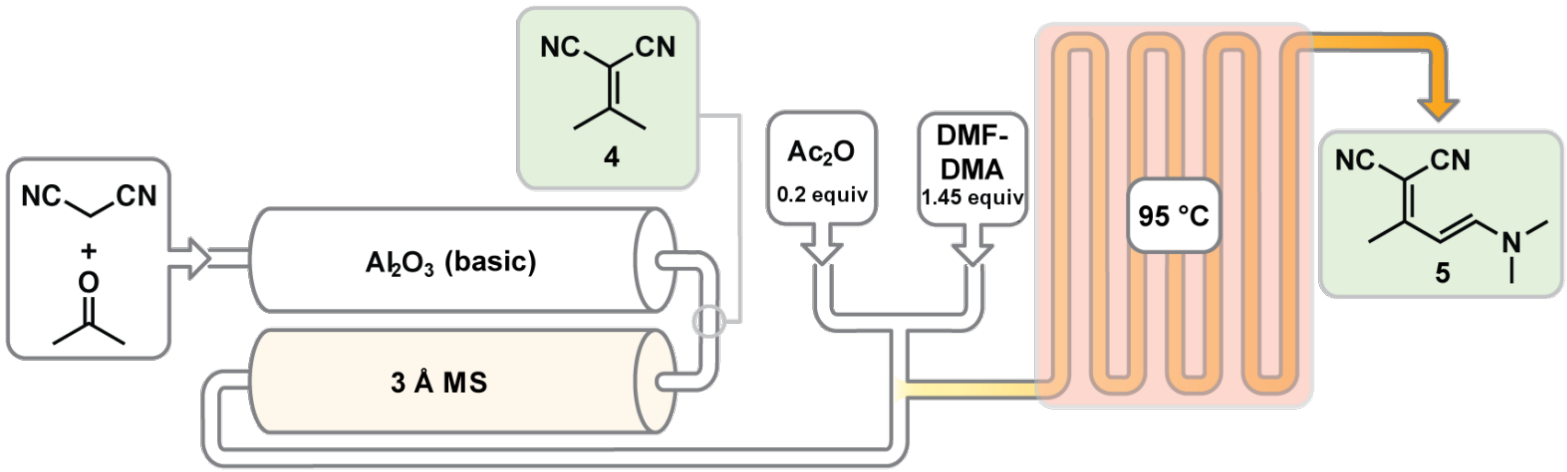
Flow scheme to produce the enamine **5** starting from acetone and malononitrile (See Supporting Information File 1 for details).

We proceeded to develop a continuous process by coupling the enamine step with the Knoevenagel condensation (Scheme 4). To achieve this, we included two columns: a packed-bed of Al_2_O_3_ to catalyze the reaction and a packed bed of 3 Å molecular sieves to absorb water before the addition of DMF-DMA (Scheme 4). The mass of each solid used (2.00 g of Al_2_O_3_ and 1.50 g molecular sieves) was chosen based on the size of the available columns. Assuming that the Knoevenagel condensation occurred primarily in the Al_2_O_3_ column, we only varied the temperature of the Al_2_O_3_ column. The Al_2_O_3_ column temperature was initially set to 25 °C which yielded 91% of **5** from acetone and malononitrile (Table 2, entry 2). We observed that the Al_2_O_3_ column reactor temperature increased during the reaction and thus examined the use of temperatures below 25 °C. Cooling the column did not provide any observable improvement (Table 2, entries 3 and 4) prompting us to examine higher temperatures. Heating the column past 25 °C increased the byproduct formation which lowered the yield (Table 2, entries 5–8). Increasing the residence time through the alumina column had no positive impact on yield (Table 2, entry 9).

**Table 2:** Screened conditions for formation of **5** starting from acetone and malononitrile.

Entry	Al_2_O_3_ column temperature (°C)	3 Å MS column temperature (°C)	Residence time of coil (min)^a^	Yield (%)^b^

1	25	20	2	NA^c^
2	25	20	4	91
3	20	25	4	91
4	10	25	4	92
5	35	20	4	88
6	50	20	4	88
7	75	20	4	84
8	95	20	4	81
9	95	20	6	81

^a^Coil temperature was 95 °C. ^b^Determined by GC analysis using mesitylene as an internal standard. ^c^The fast flow rates needed for this residence time caused the pressure of the system to exceed the maximum limit. See Scheme 4 for flow scheme.

**Figure 3:**
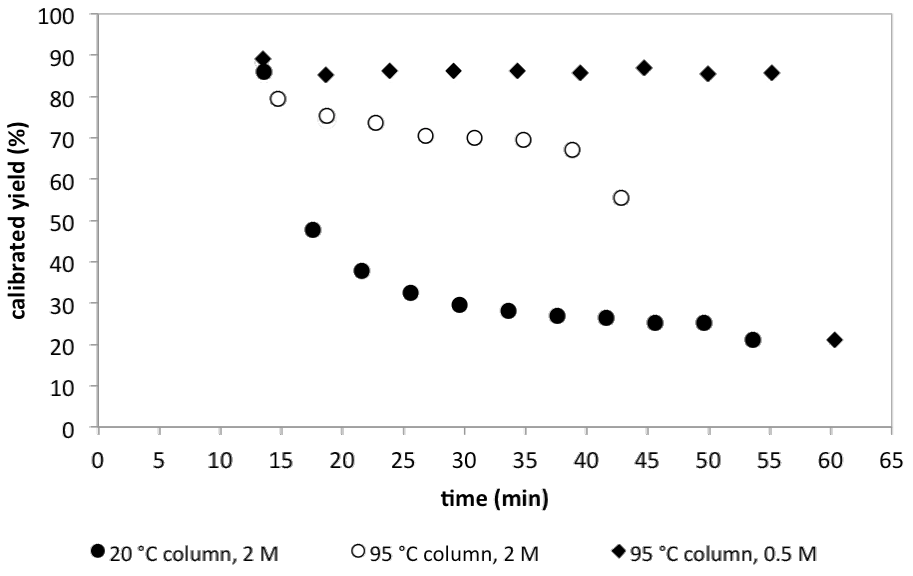
Comparing the long-term stability of the Al_2_O_3_ and 3 Å MS columns when the Al_2_O_3_ column temperature (20 °C and 95 °C) and Knoevenagel reaction concentration (2.0 M and 0.50 M) are varied. The time between 0 and 13 min was the equilibration period.

**Scheme 5:**
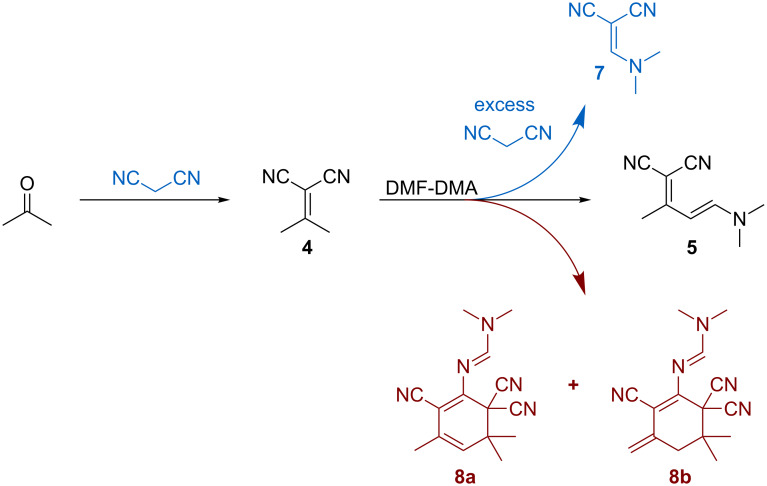
Several byproducts were observed when producing **5** starting from acetone and malononitrile. **7** is formed from excess malononitrile when the Knoevenagel reaction does not go to completion. The formation of dimers **8a** and **8b** can begin at any point during the reaction.

The successful combination of the Knoevenagel/enamine steps prompted us to evaluate the stability of this two-step system. We often find that when multisteps are combined the system stability can become an issue. To measure the stability, we ran the reaction under the optimized conditions and monitored the product distribution. When the aforementioned optimized column temperatures were used (20 °C Al_2_O_3_ column, 25 °C 3 Å MS column), the alumina column begins to fail (Figure 3). At a collection time of 12 min (Table 2, entry 3), the yield is at its maximum at 91%. However, by ~17 min, the yield drops to 48%. We speculate that at high reactant concentrations the water produced fouls the Al_2_O_3_ column. This conjecture is supported by the increased production of **7** when excess malononitrile reacts with DMF-DMA (Scheme 5). Heating the column to 95 °C allowed the Al_2_O_3_ column to remain activated longer. Despite the fact that higher alumina column temperatures result in less than optimal yields of the enamine, we examined the system stability at 95 °C. As can be seen in Figure 3, increasing the alumina column temperature provides improved stability compared to 25 °C; however, the column performance exhibits shallow decline over the first 37 min and then fails rapidly beyond 37 min. The fact that we can resurrect the column performance somewhat suggests that this stability issue can be addressed when and if this process is implemented on scale. To demonstrate that further gains in stability are possible, we examined the impact of reaction concentration on column stability.

Process chemists often seek the highest operating concentrations to reduce solvent costs. Recognizing this aspiration, we performed the aforementioned Knoevenagel reaction at 2.0 M. Considering that many process reactions run at 0.20 M, this starting concentration was high. The high starting concentration also allowed us to realize a 1.0 M reaction concentration once addition of the acetic anhydride and DMF-DMA (Scheme 4). While the higher the concentration the better, our prior efforts have revealed that packed-bed catalyst stability can rapidly decline at high concentrations while at lower concentrations can run for an extended length of time [44]. With this in mind, we lowered the Knoevenagel concentration to 0.50 M. This setup also resulted in the residence time in the Al_2_O_3_ column and 3 Å MS to reduce from 2.88 min and 2.72 min to 0.90 min and 0.85 min respectively. The faster residence time could account for less water absorption in the Al_2_O_3_ column. Because we can still hold the acetic anhydride/DMF-DMA concentrations high lowering the Knoevenagel concentration only results in the enamine concentration decreasing by factor of 1.25 (0.40 M). As shown in Figure 3, reducing the Knoevenagel to 0.50 M and heating the alumina column to 95 °C results in a dramatic improvement in system stability. The output of the enamine, however, remains similar. The reaction at a 1 M concentration would produce approximately 7 g of enamine product within the 24 min window and at a 0.4 M concentration approximately 5.8 g in the 42 min window. While we are bolstered by these improvements, we suggest that a commercial version of this process must address how to achieve long-term stability at the higher concentrations.

**Scheme 6:**
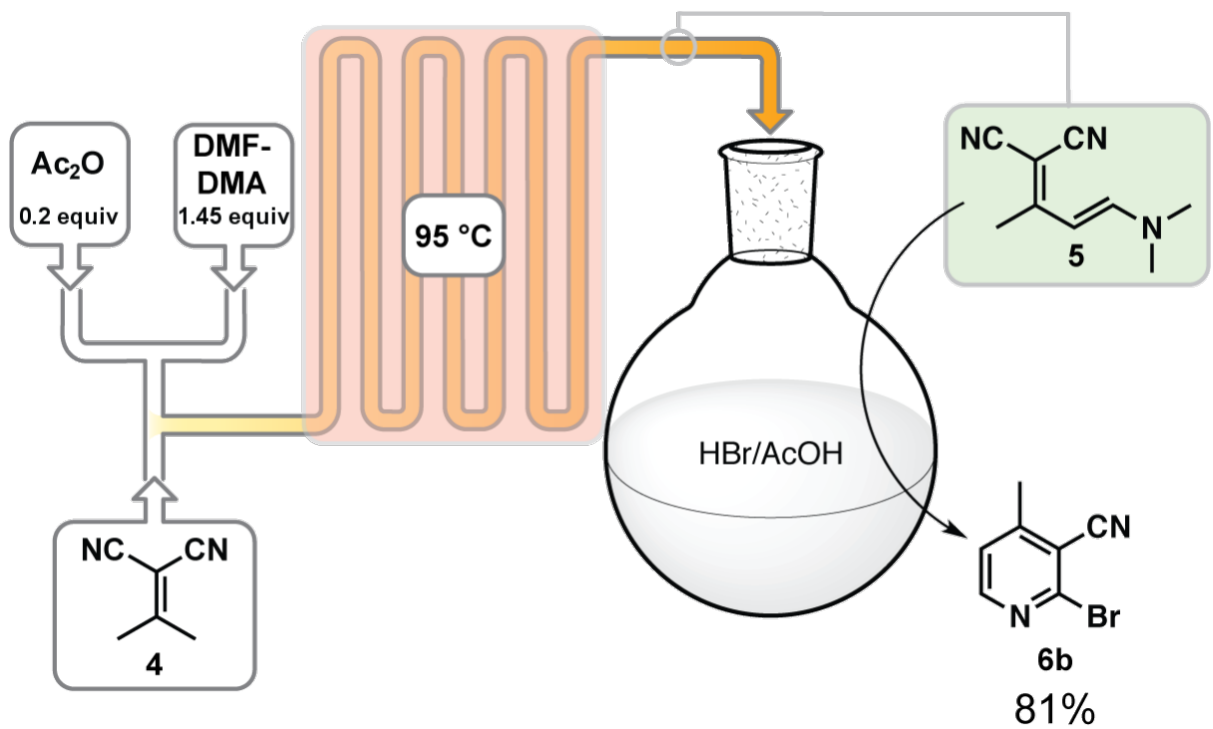
Flow scheme to produce 2-bromo-4-methylnicotinonitrile (**6b**) in 81% yield from **4** with a 1 M concentration and 2 min residence time in the coil (See Supporting Information File 1 for further details).

Creating a chemical process for an active pharmaceutical ingredient is a careful integration of chemical and regulatory challenges. While from an academic standpoint a completely continuous process provides the opportunity to advance process chemistry/technology, a new process can often require significant investment for regulatory validation. We wish to implement our technology as quickly as feasible and to do so we want to avoid potential regulatory problems. Therefore, we have opted to carry out the Pinner cyclization as terminal cyclization/crystallization step where the already validated material could be collected. To optimize the cyclization step, we combined only the enamine/cyclization steps to reduce system complexity. When we subjected 25 mL of the enamine **5** output to a solution of HBr in AcOH for 45 min (55 °C) the desired nicotinonitrile **6b** crystallized out of solution in 81% overall yield (2-steps, Scheme 6). While our intent was not to create a completely continuous process at this time, commercially available reactors that can handle strong acid are available and this step could easily be achieved in flow (for an example of an strong acid resistant reactor, see reference [38]).

**Scheme 7:**
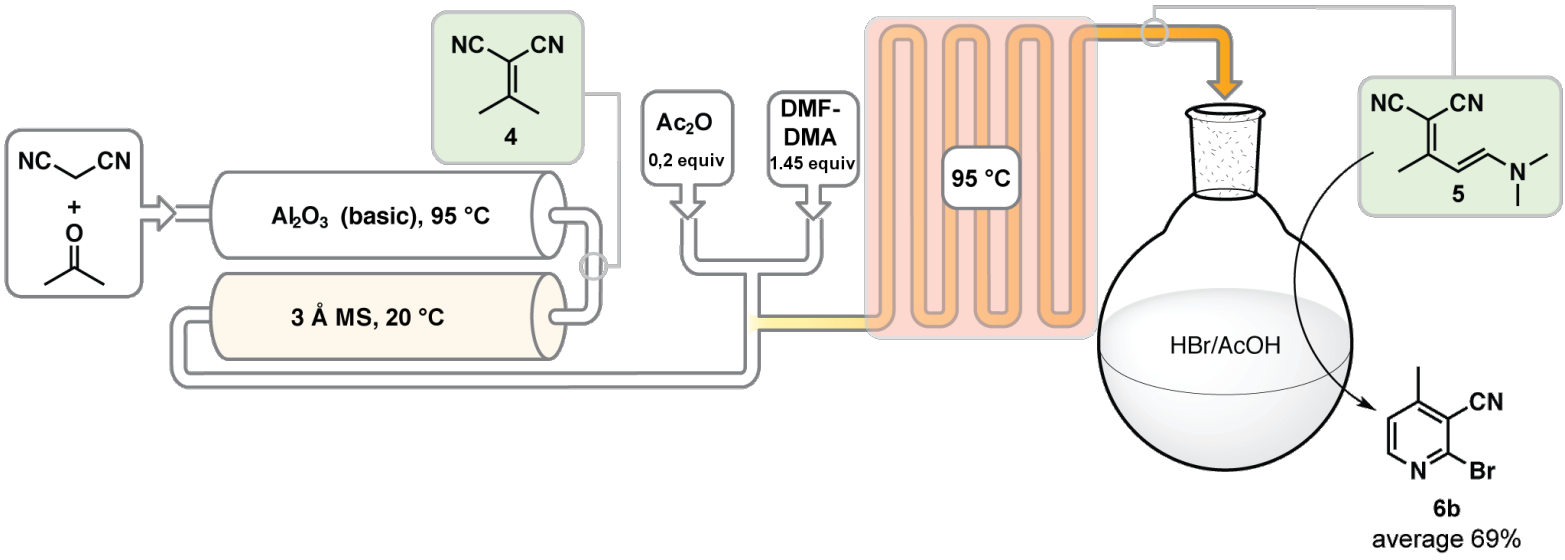
Reactor scheme for the continuous synthesis of 2-bromo-4-methylnicotinonitrile (**6b**) with an average of 69% yield. The reaction concentration within the columns was 0.50 M, while the reaction concentration in the coil was 0.40 M. The residence time in the Al_2_O_3_ column was 0.90 min, in the 3 Å MS column 0.85 min, in the coil 4 min, and the reaction time for the cyclization to occur to produce **6b** was 45 min (See Supporting Information File 1 for further details).

We completed the process by integrating all three steps together using the lower concentration Knoevenagel condensation (0.50 M) with the heated Al_2_O_3_ column at 95 °C due to its long-term stability (Scheme 7). We ran 100 mL of material through the process and after simple trituration with water 69% (5.4 g for 100 mL) of the desired 2-bromo-4-methylnicotinonitrile (**6b**) was obtained and was analytically pure as determined by elemental analysis. We have also performed the enamine cyclization using HCl instead of HBr and have produced the registered chloride in 81% yield [34].

## Conclusion

Here, we have demonstrated the semi-continuous synthesis of 2-bromo-4-methylnicotinonitrile starting from acetone and malononitrile by utilizing solid Al_2_O_3_ and 3 Å MS columns and decreasing the reaction time of the enamine formation to a matter of minutes using DCM conditions outside of normal process windows. The cyclization under Pinner conditions using the crude output from the Knoevenagel/enamine steps provides an overall yield of 69% (>88% yield per step). While the Al_2_O_3_ column is stable for a limited time (between 24 and 42 min depending on the reaction concentration used), the current solution would be to simply replace the columns throughout the production process or implement larger columns. At the current state, the amount of Al_2_O_3_ and molecular sieves needed for every one gram of 2-bromo-4-methylnicotinonitrile is 0.37 g and 0.28 g respectively. Despite the limited stability, the amount of Al_2_O_3_ used in flow to produce 5.4 g of 2-bromo-4-methylnicotinonitrile is less than the amount of Al_2_O_3_ required for the batch process (about 0.98 g is needed per 1 g of product [34]). We also predict that by replacing the alumina columns with a soluble base and a water/base separation a stable, higher concentration process can be easily achieved from our preliminary results [22,46]. Finally, we have demonstrated that from a simple acyclic precursor, 2-bromo-4-methylnicotinonitrile can be achieved in a three-unit operation process yielding high purity crystalline materials. The chloride product is already registered and suggests that this strategy could be implemented in existing nevirapine processes.

## Supporting Information

The Supporting Information describes synthesis and characterization data of all substances given in this article, reactor setup, operational details and screening conditions.

File 1 Experimantal section.
