# Supplementary material for: Investigating the continuous synthesis of a nicotinonitrile precursor to nevirapine
Source: Beilstein J Org Chem. 2013 Nov 20;9:2570–8. doi: 10.3762/bjoc.9.292 (PMC3869350; doi:10.3762/bjoc.9.292)

Supporting Information

for

Investigating the continuous synthesis of a nicotinonitrile precursor to nevirapine

Ashley R. Longstreet[1], Suzanne M. Opalka[1], Brian S. Campbell[1], B. Frank Gupton[2] and D. Tyler McQuade*[1]

Address: [1]Department of Chemistry and Biochemistry, Florida State University, 95 Chieftan Way, Tallahassee, FL 32306, United States and [2]Department of Chemistry, Virginia Commonwealth University, 1001 West Main Street, P.O. Box 842006 Richmond, Virginia 23284, United States

Email: D. Tyler McQuade* - mcquade@chem.fsu.edu

* Corresponding author

Experimental Section

Contents	Page
1. General information	S2
2. Batch preparation of reagents and standards	S2–S3
3. Reactor setup and procedure for enamine reaction temperature screen	S3–S4
4. Reactor setup and procedure for enamine reaction concentration screen	S4
5. Continuous synthesis of enamine from acetone and malononitrile	S5–S6
6. Reactor setup and procedure for long-term stability experiments	S6-S7
7. Continuous synthesis of 2-bromo-4-methylnicotinonitrile from isopropylidenemalononitrile	S7–S8
8. Continuous synthesis of 2-bromo-4-methylnicotinonitrile from acetone and malononitrile	S8–S9
9. Characterization of 2-bromo-4-methylnicotinonitrile	S9
10. References	S9
11. ^{1}H and ^{13}C NMR spectra of 2-bromo-4-methylnicotinonitrile	S10

1. General information

Malononitrile was purified by recrystallization in ethanol prior to use. All other commercially available reagents were used without further purification. Proton nuclear magnetic resonance (^{1}H NMR) spectra and carbon nuclear magnetic resonance (^{13}C NMR) spectra were recorded on a Bruker 600 MHz spectrometer. Chemical shifts for protons are reported in parts per million downfield from tetramethylsilane. Chemical shifts for carbon are reported in parts per million downfield from tetramethylsilane or referenced to residual solvent. Data are represented as follows: chemical shift, multiplicity (br = broad, s = singlet, d = doublet, t = triplet, q = quartet, m = multiplet), coupling constants in Hertz (Hz), integration.

Gas chromatographic analyses were performed using a GC equipped with a flame ionization detector (FID) and GC column with 30 m length, 0.32 mm inner diameter, and 0.25 µm film thickness. The method was as followed. The inlet was set to 250 ºC with a split ratio of 10:1. The temperature gradient used was 60 ºC initially with a 1 min hold time, then heated to 100 ºC with a 5 ºC/min ramp, then lastly heated to 250 ºC with a 50 ºC/min ramp and a 8 min hold time. Flow rate was set to 4 mL/min. The enamine (**5**) did degrade slightly on the column, so GC yields obtained are within error. Mesitylene was used as the internal standard.

Reactions were performed with a commercially available Vapourtec R series reactor controlled by FlowCommander™ software. Aluminum oxide (activated basic, Brockmann I) and powdered 3 Å molecular sieves (MS) were packed into a glass Omnifit column (6.6 mm diameter, 100 mm length) fitted with Vapourtec end caps and PTFE frits. All tubing and fittings were supplied with the reactor, but the tubing was standard 1.00 mm bore PFA and standard PTFE fittings.

2. Batch preparation of reagents and standards

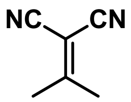

4

Isopropylidenemalononitrile (4). Prepared according to the previously reported procedure with some modifications [1]. Malononitrile (11.69 g, 177 mmol, 1 equiv) was dissolved in HPLC grade acetone (13.0 mL, 177 mmol, 1 equiv). To this was added $CHCl_3$ (100 mL). Al_2O_3 (activated basic, Brockmann I) (25.0 g, 245 mmol, 1.39 equiv) was added slowly to the stirred solution to prevent overheating. After the addition, the reaction stirred for an additional 1 h. The Al_2O_3 was then filtered off by vacuum filtration and rinsed with DCM (150 mL). The resulting filtrate was concentrated in vacuo, then short-path distillation (60 ºC, 300–400 mTorr) was performed to yield **4** as a colorless oil (16.53 g, 88%). Contained traces of malononitrile (<1.0 wt %). ^{1}H NMR: (600 MHz, $CDCl_3$) δ 2.32 (s, 6H) ppm. ^{13}C NMR: (151 MHz, $CDCl_3$) δ 178.8, 111.8 (2C), 86.1, 24.5 (2C) ppm. Spectra were in accordance with those described in the literature [1].

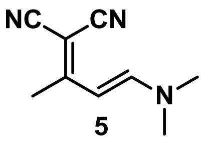

2-(4-(Dimethylamino)but-3-en-2-ylidene)malononitrile (5). To a round-bottom flask containing isopropylidenemalononitrile (**4**) (2.12 g, 20 mmol, 1 equiv) was added dry toluene (20 mL) and acetic anhydride (0.38 mL, 4 mmol, 0.2 equiv). The flask was placed in a preheated oil bath at 45 ºC, then *N*,*N*-dimethylformamide dimethyl acetal (DMF-DMA) (3.20 mL, 24 mmol, 1.2 equiv) was added to the reaction dropwise while stirring. The solution instantly turned yellow, and an orange solid began to form within 5 minutes of the DMF-DMA addition. After 1 h, the reaction was removed from the heat and hexanes (30 mL) was added. After cooling the solution, the orange solid was collected by vacuum filtration, rinsed with a cooled 50:50 toluene/hexanes solution, and dried under vacuum to yield **5** (2.97 g, 92%). ^{1}H NMR: (600 MHz, $CDCl_3$) δ 7.26 (d, J = 12.7 Hz, 1H), 5.62 (d, J = 12.6 Hz, 1H), 3.23 (s, 3H), 2.99 (s, 3H), 2.23 (s, 3H) ppm. ^{13}C NMR: (151 MHz, $CDCl_3$) δ 168.2, 152.4, 116.9, 116.1, 97.1, 65.5, 45.8, 37.5, 17.2 ppm. Spectra were in accordance with those described in the literature [2].

3. Reactor setup and procedure for enamine reaction temperature screen

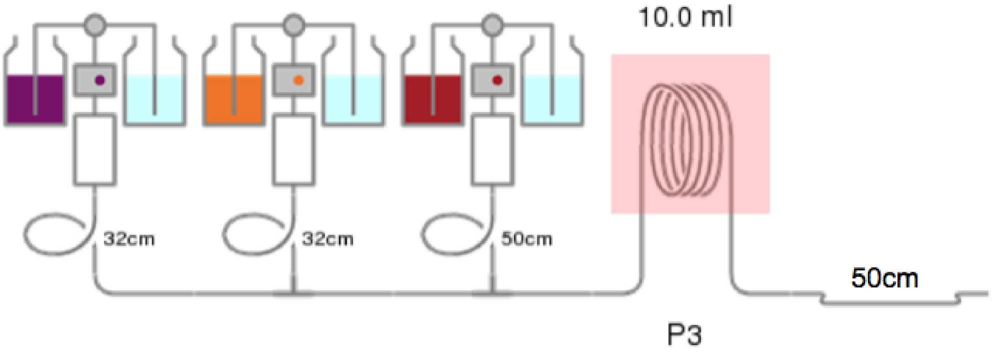

Reactor setup: A stock solution of isopropylidenemalononitrile (**4**) (0.2 M) in DCM was prepared for pump A (purple), a stock solution of DMF-DMA (0.48 M) and mesitylene (0.04 M) in DCM was prepared for pump B (orange), and a stock solution of acetic anhydride (0.08 M) in DCM was prepared for pump C (red). Each pump was connected to drop tubing (32 cm, 32 cm, and 50 cm respectively), which were connected together before entering the heated reactor with a four-port mixer. The heated reactor contained tubing with a 10 mL volume. Additional tubing was connected outside of the reactor (50 cm), and a 250 psi backpressure regulator (BPR) (not shown) was placed at the end of this tubing. After the BPR, additional tubing was present before reaching the fraction collector to dispense the sample.

Procedure: For each reaction, the residence time inside the heated reactor was set to 2 min with flow rates for pump A set to 2.50 mL/min, pump B 1.25 mL/min, and pump C 1.25 mL/min (2:1:1 ratio). The reagents were pumped through the preheated heated reactor heated to temperatures between 30 to 130 ºC. The equilibration period was

determined using the FlowCommander™ software. Upon collection of a 2.5 mL sample, a sample to be submitted to the GC was obtained and quenched with 5 µL of water.

4. Reactor setup and procedure for enamine reaction concentration screen

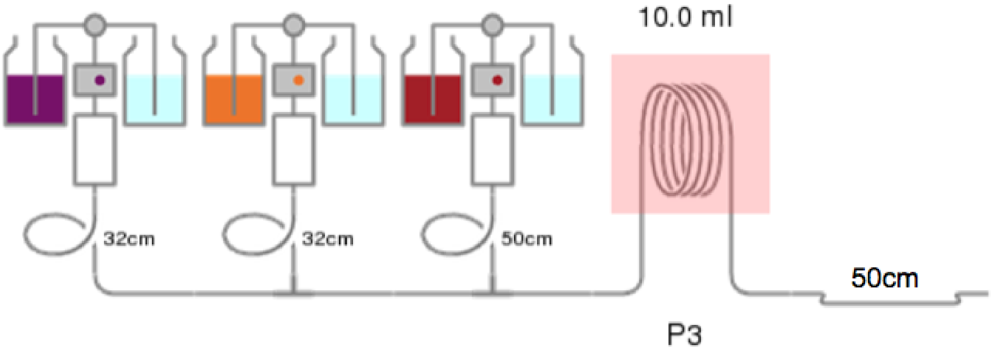

Reactor setup: A stock solution of isopropylidenemalononitrile (**4**) (2 M) and mesitylene (0.2 M) in DCM was prepared for pump A (purple), and a stock solution of DMF-DMA (2.4 M) and acetic anhydride (0.4 M) in DCM was prepared for pump B (orange). Pump C (red) was used to pump DCM to dilute each of the reactions to the appropriate concentration. Each pump was connected to drop tubing (32 cm, 32 cm, and 50 cm respectively), which were connected together by a four-port mixer before entering the heated reactor. The heated reactor contained tubing with a 10 mL volume. Additional tubing was connected outside of the reactor (50 cm), and a 100 psi BPR (not shown) as placed at the end of this tubing. After the BPR, additional tubing was present before reaching the fraction collector to dispense the sample.

Procedure: For each reaction, the reagents were pumped through heated reactor preheated to 95 ºC with a 2 min residence time. The equilibration period was determined using the FlowCommander™ software. Upon collection of a 2.5 mL sample, a sample to be submitted to the GC was obtained and quenched with 5 µL of water. The pump flow rates for each reaction were as followed:

Reaction concentration (M)	Reagent ratios (A:B:C)	Pump A flow rate (mL/min)	Pump B flow rate (mL/min)	Pump C flow rate (mL/min)
0.2	1:1:8	0.500	0.500	4.00
0.4	1:1:3	1.00	1.00	3.00
0.6	1:1:1.33	1.50	1.50	2.00
0.8	1:1:0.5	2.00	2.00	1.00
0.98	1:1:0.05	2.45	2.45	0.100

5. Continuous synthesis of enamine from acetone and malononitrile

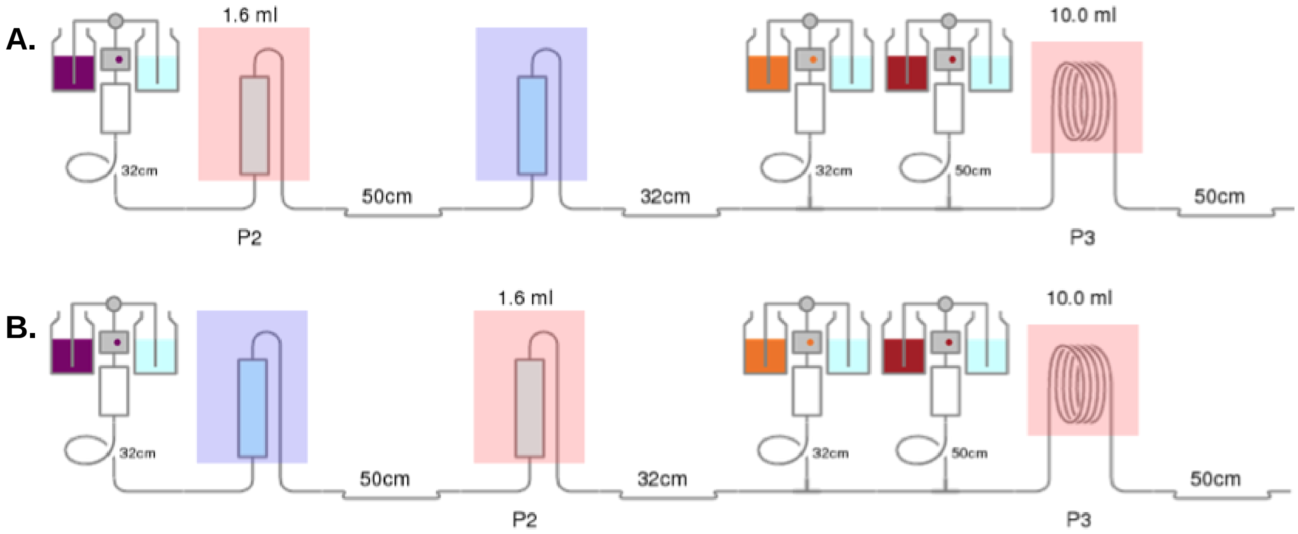

Reactor A setup: A stock solution of acetone (2 M) and malononitrile (2 M) in DCM was prepared for pump A (purple), a stock solution of DMF-DMA (5.8 M) and mesitylene (0.4 M) in DCM was prepared for pump B (orange), and a stock solution of acetic anhydride (0.8 M) in DCM was prepared for pump C (red). 32 cm of drop tubing from pump A was connected to the bottom of the first packed bed column of Al_2O_3 in a heated reactor. 50 cm of additional tubing was used to connect the top of the first column to the bottom of the second packed bed column of 3 Å MS in a cooled reactor set to 20 ºC. The top of the second column was connected to 32 cm of additional tubing, which met the 32 cm drop tubing from pump B and 50 cm drop tubing from pump C at a four-port mixer. The mixture then entered the coil of tubing (10 mL volume) in a heated reactor at 95 ºC. Additional tubing was connected outside of the reactor (50 cm), and a 100 psi BPR (not shown) as placed at the end of this tubing. After the BPR, additional tubing was present before reaching the fraction collector to dispense the sample.

Reactor B setup: Refer to reactor setup A with the following modifications. 32 cm of drop tubing from pump A was connected to the bottom of the packed bed of Al_2O_3 in a cooled reactor. 50 cm of additional tubing was used to connect the top of the first column to the bottom of the packed bed of 3 Å MS in a heated reactor set to 25 ºC. The top of the second column was connected to 32 cm of additional tubing, which met the 32 cm drop tubing from pump B and 50 cm drop tubing from pump C at a four-port mixer.

Column preparation: For each reaction, fresh columns were prepared. Reagents used were from recently opened containers and were not activated further prior to use. The first column was packed with Al_2O_3 (2.00 g). To ensure the column was packed well, the column was tapped on the bench top several times to allow the granules to settle, and

then the column was packed further by tightening the endcap. The second column was packed with powdered 3 Å MS (1.50 g). The column was tapped on the bench top to allow the powder to settle, and then the column was packed further by tightening the endcap. It is important not to pack the column too tight. A column packed too tight would cause the pressure of the system to be too high. The last step in ensuring the columns were packed well was by passing dry DCM through each at 2.25 mL/min for 5 min. Columns that not prepared using the following would not produce the desired results.

Procedure: When the Al_2O_3 column temperature was ≥25 ºC, reactor setup A was used, and at temperatures ≤20 ºC, reactor setup B was used. The reagents were mixed in a 2:1:1 ratio (pump A:B:C). Both columns and the coil were preheated or precooled to the appropriate temperature before the reaction took place. The equilibration period was determined using the FlowCommander™ software. Upon collection of a 2.5 mL sample at 12 min (22 min with a 6 min coil residence time), a sample to be submitted to the GC was obtained and quenched with 5 µL of water. The flow rates used dependent on the residence time of the coil are indicated below:

Coil residence time (min)	Pump A flow rate (mL/min)	Pump B flow rate (mL/min)	Pump C flow rate (mL/min)
2	2.50	1.25	1.25
4	1.25	0.625	0.625
6	0.833	0.417	0.417

6. Reactor setup and procedure for long-term stability experiments

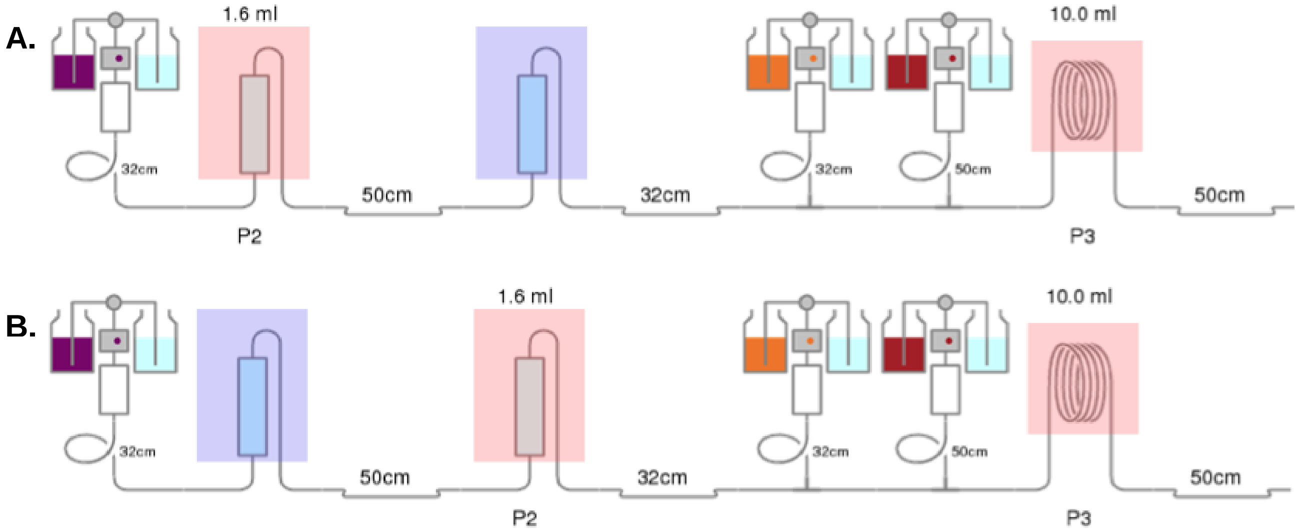

Reactor A setup: Refer to reactor A setup in section 6 with the following modifications. For pump A, either a 0.5 M (for 0.5 M column reaction concentration) or 2 M (for 2 M

column reaction concentration) stock solution of acetone and malononitrile were used. The Al_2O_3 column was heated to 95 ºC.

Reactor B setup: Refer to reactor B setup in section 6 with the following modifications. The Al_2O_3 column was cooled to 20 ºC.

Column preparation: Refer to column preparation in section 6.

Procedure: Both columns and the coil were preheated or precooled to the appropriate temperature before the reaction took place. After the equilibration period, determined using the FlowCommander™ software, 1 mL samples were taken every 4.01 min when the reaction concentration in the columns was 2 M and every 5.21 min when the reaction concentration in the columns was 0.5 M. Up to 11 samples were collected unless the pressure of the system exceeded the maximum limit before all samples were collected. Upon collection of the 1 mL sample, a sample to be submitted to the GC was obtained and quenched with 5 µL of water. The flow rates and mixing ratios for each reaction concentration for a 4 min coil residence time were as followed:

Reaction concentration within the columns (M)	Reaction concentration within the coil (M)	Reagent ratios (A:B:C)	Pump A flow rate (mL/min)	Pump B flow rate (mL/min)	Pump C flow rate (mL/min)
2	1	2:1:1	1.25	0.625	0.625
0.5	0.4	8:1:1	2.00	0.250	0.250

7. Continuous synthesis of 2-bromo-4-methylnicotinonitrile from isopropylidenemalononitrile

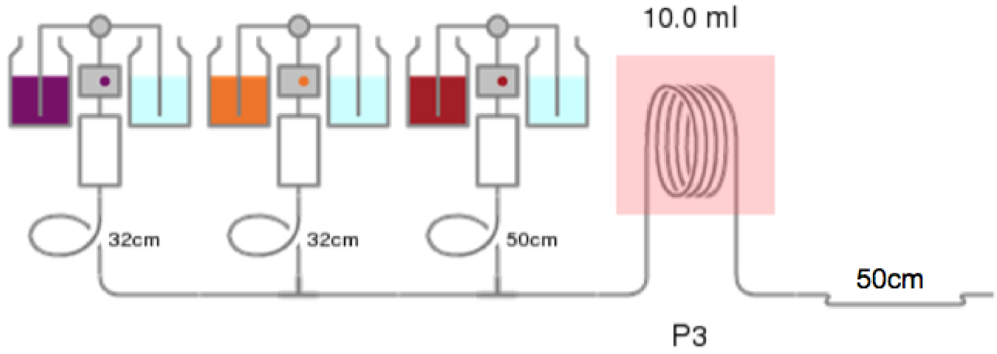

Reactor setup: A stock solution of isopropylidenemalononitrile (**4**) (3 M) in DCM was prepared for pump A (purple), a stock solution of DMF-DMA (4.2 M) and mesitylene (0.3 M) in DCM was prepared for pump B (orange), and a stock solution of acetic anhydride (0.6 M) in DCM was prepared for pump C (red). Each pump was connected to drop tubing (32 cm, 32 cm, and 50 cm respectively), which were connected together before

entering the heated reactor with a four-port mixer. The heated reactor contained tubing with a 10 mL volume. Additional tubing was connected outside of the reactor (50 cm), and a 100 psi BPR (not shown) as placed at the end of this tubing. After the BPR, additional tubing was present before reaching the fraction collector to dispense the sample.

Procedure: The reagents were pumped through heated reactor preheated to 95 ºC with a 2 min residence time. The flow rate of each pump was set to 1.66 mL/min. The equilibration period was determined using the FlowCommander™ software. After the equilibration period, 25 mL of the reaction mixture was collected and immediately transferred to a round-bottom flask with AcOH (15 mL). A 33 wt % HBr in AcOH solution (27.5 mL) was added dropwise to the mixture. Once the addition was complete, a condenser was attached to the flask and the reaction was heated to 55 ºC for 45 min. Upon cooling the reaction to room temperature, DCM was removed in vacuo. 400 mL of water was added to the remaining solution to cause the product to precipitate out. The solution was cooled before the product was collected by vacuum filtration and dried under high vacuum over P_2O_5 to yield **6b** as an orange solid (3.97 g, 81%).

8. Continuous synthesis of 2-bromo-4-methylnicotinonitrile

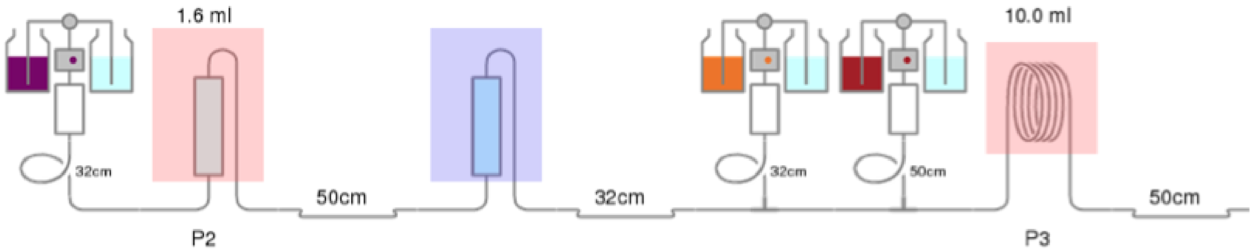

Reactor setup: Refer to reactor A setup in section 6 with the following modifications. For pump A, a stock solution of acetone (0.5 M) and malononitrile (0.5 M) was used. The Al_2O_3 column was heated to 95 ºC.

Column preparation: Refer to column preparation in section 6.

Procedure: Both columns and the coil were preheated or precooled to the appropriate temperature prior to use. The flow rates for pumps A, B, and C were set to 2.00, 0.250, and 0.250 mL/min respectively for a 4 min residence time in the heated coil. After the equilibration period, determined using the FlowCommander™ software, 6 × 18 mL samples were collected. Immediately after collection, 2 × 15 mL of the reaction mixture from 2 vials were charged to a mixture of AcOH (7.2 mL) and a 33 wt% HBr in AcOH solution (13.2 mL) in a round-bottom flask via syringe while stirring vigorously (three reactions from one reactor run were performed in total). A condenser was attached to the flask and the reaction was heated to 55 ºC for 45 min. Upon cooling the reaction to room temperature, DCM was removed in vacuo. 200 mL of water was added to the

remaining solution to cause the product to precipitate out. The solution was cooled before the product was collected by vacuum filtration and dried under high vacuum over P_2O_5 to yield **6b** as an orange solid (first two fractions: 1.61 g, 68%, second two fractions: 1.65 g, 70%, third two fractions fraction: 1.62 g, 69%). To remove the orange color, the product was passed through a silica plug using DCM as the eluent. Upon removal of the solvent in vacuo, 95% of **6b** was recovered as an off-white solid.

9. Characterization of 2-bromo-4-methylnicotinonitrile

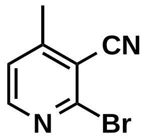

6b

2-Bromo-4-methylnicotinonitrile (6b). Spectra and elemental analysis obtained before removal of the orange discoloration. ^{1}H NMR: (600 MHz, $CDCl_3$) δ 8.41 (d, *J* = 5.1 Hz, 1H), 7.29 (d, *J* = 5.1 Hz, 1H), 2.62 (s, 3H) ppm. ^{13}C NMR: (151 MHz, $CDCl_3$) δ 154.7, 152.0, 144.3, 124.0, 115.1, 114.8, 21.0 ppm. Anal. Calcd. for $C_7H_5BrN_2$: C: 42.67; H: 2.56; N: 14.22. Found: C: 42.42; H: 2.63; N: 14.11.

10. References

[1] Broman, S. L.; Petersen, A. U.; Tortzen, C. G.; Vibenholt, J.; Bond, A. D.; Nielsen, M. B. *Org. Lett.* **2011**, *14*, 318–321.

[2] Ege, G.; Frey, H. O.; Schuck, E. *Synthesis* **1979**, 376–378.

11. ^{1}H and ^{13}C NMR spectra of 2-bromo-4-methylnicotinonitrile

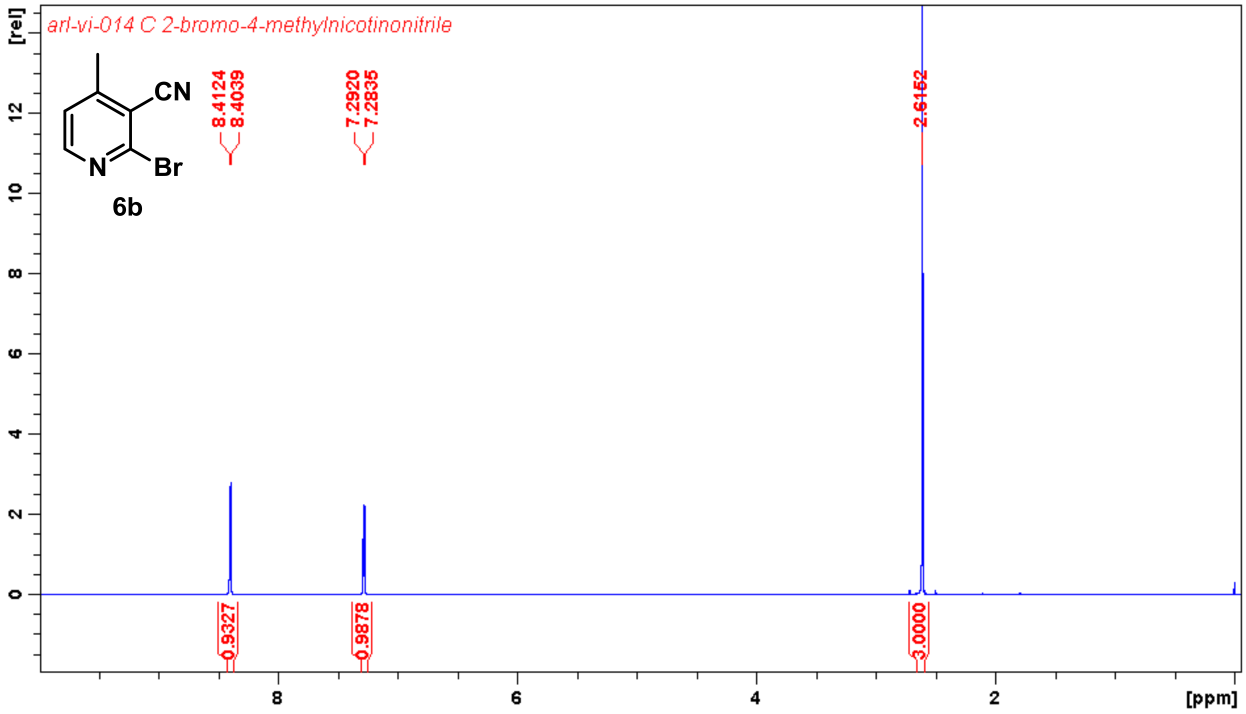

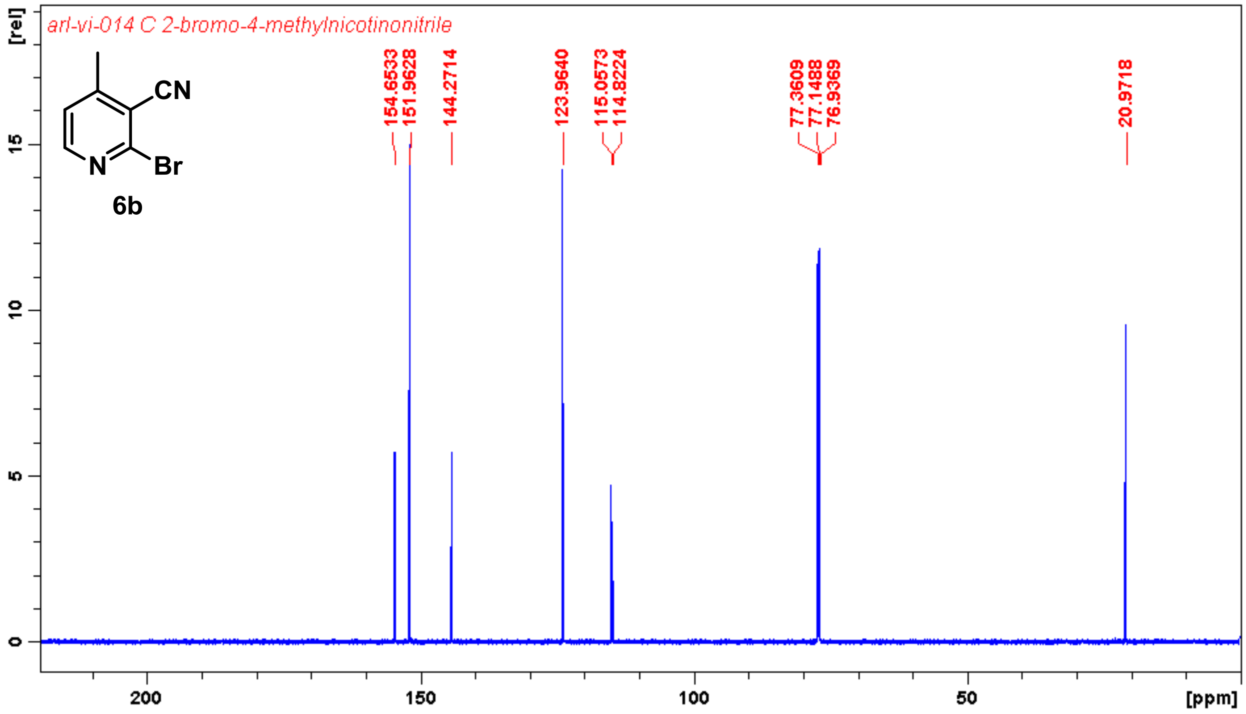

Supplement: File 1 — Experimantal section. [file Beilstein_J_Org_Chem-09-2570-s001.pdf]
